# Immunotherapy in Non-Small Cell Lung Cancer: Shifting Prognostic Paradigms

**DOI:** 10.3390/jcm7060151

**Published:** 2018-06-14

**Authors:** Megan B. Barnet, Wendy A. Cooper, Michael J. Boyer, Steven Kao

**Affiliations:** 1 The Garvan Institute of Medical Research, Darlinghurst, NSW 2010, Australia; 2 Graduate Research School, University of New South Wales, Sydney, NSW 2052, Australia; 3 Sydney Medical School, The University of Sydney, Camperdown, NSW 2006, Australia; wendy.cooper@health.nsw.gov.au (W.A.C.); michael.boyer@lh.org.au (M.J.B.); steven.kao@lh.org.au (S.K.); 4 Royal Prince Alfred Hospital, Camperdown, NSW 2006, Australia; 5 School of Medicine, Western Sydney University, Penrith, NSW 2751, Australia; 6 The Chris O’Brien Lifehouse, Camperdown, NSW 2006, Australia

**Keywords:** non-small cell lung cancer, immunotherapy, checkpoint inhibitors

## Abstract

Immune checkpoint inhibitors have shown efficacy in the treatment of non-small cell lung cancer (NSCLC) in the adjuvant, first- and subsequent-line settings. In metastatic disease, they provide hope of durable response where “best-case” scenario has long been inadequate. This progress has highlighted the immunogenic nature of NSCLC and invigorated research into immunotherapy in the field. In this review we consider the foundations of immunotherapy in NSCLC, canvass the current research and summarise the evidence guiding clinical practice.

## 1. Introduction

Recognition of the role of immunity in health and disease is not new, however our understanding of its complexity has evolved significantly. From a clinical observation of the possible therapeutic effect of infection, to the targeting of specific immune pathways, immunotherapy has followed a circuitous route to enter the mainstream of oncology treatments.

Checkpoint inhibitors (CPI) are a relatively new class of drugs that prevent or reverse the pathological dampening of a host’s immune response to cancer. The most clinically advanced CPIs are monoclonal antibodies (mAb) that target the programmed cell death-1/programmed cell death-ligand 1 (PD-1/PD-L1) and cytotoxic T-lymphocyte antigen 4 (CTLA-4) pathways. By binding PD-1/PD-L1 and/or CTLA-4, this subset of CPIs can prevent immune suppression and facilitate immune stimulation respectively. The success of anti-PD-1/PD-L1 and anti-CTLA-4 agents has reinvigorated immunotherapy research and prompted a wave of clinical trials combining the drugs with other therapies.

Historically, immunotherapy has shown mixed success in solid organ cancer, punctuated by successes such as intra-vesical Bacillus Calmette-Guerin (BCG) and now CPIs. Within non-small cell lung cancer (NSCLC), CPIs now have an established role in metastatic disease and an emerging role in the adjuvant setting. This review will discuss the evolution of immunotherapy in oncology and review the major clinical trials informing current practice in NSCLC.

## 2. Immunotherapy Has Evolved Over Centuries

### 2.1. Therapeutic Infection and Cytokines

William Coley originally observed in the late 1800s that inducing erysipelas by inoculation with a mixture containing *S. pyogenes* and *S. marcasens* in patients with sarcoma had a “curative effect” in some cases [1] This by-product of inflammation was utilised in the contemporaneous practice of pyrotherapy-fever as treatment for disease-notably by Julius Wagner-Jauregg, who won a Nobel prize for his research on malaria as a treatment for neurosyphilis [2]. Development of chemotherapy and penicillin made these methods redundant, but attempts at reproducing the anti-cancer effect induced by inflammation continued.

Murine models through the 1900s demonstrated tumour regression following bacterial endotoxin inoculation and, furthermore, tumour regression in animals receiving serum only from inoculated animals [3,4]. Host cells were shown to excrete a crucial factor in this reaction, coined “tumour necrosis factor” (TNF), which mimicked the toxic effect of endotoxin [5]. Research into TNF revealed a network of related ligands and receptors with broad-ranging immune roles, stimulating further research into this field [6]. Notable examples of cytokines used with some clinical success include IL2 and IFNα, US Food and Drug Administration (FDA) approved for metastatic melanoma/renal cell carcinoma and adjuvant treatment in stage III melanoma respectively. The most enduring infection-based immunotherapy is Bacillus Calmette-Guerin (BCG); which was introduced in 1976 and has persisted in treatment of localised bladder cancer for over 40 years [7].

### 2.2. Monoclonal Antibodies

Development of targeted therapies stemmed from improved understanding of molecular pathways and the capability to engineer drugs. In 1975, Kohler and Milstein outlined a technique to generate specific antibody, involving fusion of B-lymphocytes from an immunised murine host with an immortal myeloma cell line, then isolating specific-antibody producing clones [8]. Technical advances then enabled human chimerism, reducing rates of allergy and anti-drug antibody formation [9]. Flagship immune-targeted chimeric monoclonal antibodies (mAbs) such as rituximab (anti-CD20) and infliximab (anti-TNFα) were licensed in the late 1990s and remain in use today.

Co-stimulatory and co-inhibitory signals play a vital role in immune activation and containment, and are collectively called “checkpoints”. The recognition that malignant immune escape was facilitated, in part, by tumour up-regulation of inhibitory checkpoints fuelled research into therapeutic blockade of these signals. The two best-characterised inhibitory checkpoints are CTLA-4 and PD-1. CTLA-4 is expressed on regulatory T cells constitutively and on conventional T cells early in activation. It is homologous with the co-stimulatory T-cell receptor CD28, and competitively binds its ligands B7-1 (CD80) and B7-2 (CD86), thereby blocking the requisite “2nd signal” to stimulate T-cell expansion. PD-1 is also expressed during T cell activation and serves as a negative feedback mechanism to curtail T-cell expansion. Ligation of PD-1 by its ligands, PD-L1 or PD-L2, initiates inhibitory signals that result in de-phosphorylation (inactivation) of stimulatory effector molecules induced by T-cell receptor (TCR) and CD28 ligation. CTLA-4 was the first inhibitory receptor to be targeted in clinical trials, with phase I data from the blocking antibody “MDX-CTLA4” (ipilimumab) showing clinical activity in 2003, but lacking supportive phase III evidence until 2010 [10,11]. Simultaneously, data was emerging around a second mAb targeting PD-1, “MDX-1106” (nivolumab), with pre-clinical suggestion of reduced toxicity compared with ipilimumab [12].

In the short years since, there has been a relative explosion of checkpoint inhibitor therapy within oncology. For PD-1/PD-L1 mAbs alone, FDA-approved settings now include melanoma, NSCLC, head and neck squamous cell carcinoma, urothelial carcinoma, clear cell renal cell carcinoma, hepatocellular carcinoma, Merkel Cell Carcinoma, mismatch repair (MMR)-deficient cancer of any origin and Hodgkin Lymphoma (www.fda.gov).

### 2.3. Adoptive Cell Therapy

Adoptive cell therapy relies on ex-vivo manipulation of T cells to accomplish clonal expansion of anti-tumour effector T cells. This can be done either by isolation of tumour infiltrating lymphocytes (TILs) and reinfusion after expansion, or synthetic manipulation of TCRs ex vivo to form chimeric antigen receptors (CARs). CAR-T cells are encoded with a viral vector, the machinery of which allows the foreign RNA to reverse-transcribe into the DNA of host T cells and integrate into the genome. Subsequent generation drugs improved response rates by incorporating co-stimulatory receptors (often CD28 or 4-1BB). The cells are then cultured and re-infused following lymphodepletion therapy, with great risk of toxicity in the form of cytokine release and macrophage activation syndromes.

CAR-T therapy has shown most effect in select B cell malignancies, though many trials are active in solid tumours [13]. Homogenous surface protein expression, CD19 in the case of B-cell acute lympoblastic leukaemia (ALL), provides an ideal target for the clonal TCR of CAR-T cells. A major obstacle to uptake is cost—the first FDA—approved compound for B-cell ALL, Kymriah, has a list price of US$475,000 for the one-off treatment. Further issues with transition of CAR-T’s to solid organ cancers include an immunosuppressive tumour microenvironment (TME), high antigenic heterogeneity, and tendency for known tumour-associated antigens (TAAs) to be shared with other tissues, increasing risk of toxicity [14].

### 2.4. Tumour Vaccines

Therapeutic vaccination aims to strengthen a patient’s own anti-tumour immune response against a broad range of TAA’s. Categories of vaccines include cell-based (tumour or immune), peptide-based and genetic (DNA, RNA or viral) [15]. Cell-based vaccines utilising antigen-presenting cells (APCs) such as dendritic cells (DCs) are the most clinically advanced of these. DC vaccines are built on the capacity to load these sentinel immune cells with specific antigen ex vivo, creating a stimulant of innate and adaptive immunity to the desired antigen on re-infusion [15]. Sipuleucel-T remains the first and only therapeutic vaccination to gain FDA approval in 2010, and is generated by incubating peripheral blood mononuclear cells with a prostate antigen, prostatic acid phosphatase, fused to GM-CSF to stimulate proliferation [16].

## 3. Immunotherapy in Non-Small Cell Lung Cancer

NSCLC is ideally placed to benefit from immunotherapy, based on the relatively high prevalence of somatic protein mutations in the cancer genome [17]. Somatic mutations can occur as a result of intrinsic factors such as defective DNA repair, or extrinsic factors such as mutagen exposure—as is often the case in lung cancer, which remains a predominantly smoking-related disease. Tumour mutational burden (TMB), a measure of somatic mutations, is relatively high in NSCLC compared with other malignancies and has been shown to be an independent predictor of response to CPIs [17,18].

To date, mAb CPIs are the only form of immunotherapy used as standard treatment in NSCLC; both as single agents or in combination with other treatments. Despite promising early phase results for vaccines, phase III trials have mostly been negative (the exception, racotumomab, is discussed below)—perhaps due to insufficient power, based on the improvement in OS in the pooled meta-analysis [19]. Adoptive cell therapy has been investigated in pre-clinical to phase II settings, however is under-represented in phase III trials due to lack of efficacy and/or unacceptable toxicity [14,20]. There are no phase III adoptive cell therapy trials in lung cancer currently registered with clinicaltrials.gov.

A discussion of completed and upcoming phase III trials is given below, with emphasis on practice changing trials for FDA-approved drugs. These are summarised in Table 1 and Table 2.

### 3.1. Metastatic Disease—Second Line

The flagship phase III trials showing efficacy for CPIs in squamous and non-squamous NSCLC respectively were CheckMate-017 and CheckMate-057 [21,22]. CheckMate-017, published in July 2015, showed improved median overall survival (OS) (9.2 vs. 6.0 months), response rate (20% vs. 9%) and median progression free survival (PFS) (3.5 vs. 2.8 months) for nivolumab compared with docetaxel as treatment for squamous cell NSCLC in the second line setting [21]. Following this CheckMate-057, in non-squamous NSCLC, showed similar benefit compared with docetaxel, with OS 12.2 vs. 9.4 months and response rate 19% vs. 12% [22]. Notably, median PFS in this trial did not show benefit (2.3 months vs. 4.2 months), though the rate of PFS at one year was significantly better (19% vs. 8%) [22]. This is an important learning point in immunotherapy compared with conventional trials; median PFS has proved a poor surrogate for OS, with closer statistical correlation found with landmark values such as six-month PFS [23,24,25,26].

Toxicity of nivolumab was favourable in both of these trials, as it was in the corresponding trials of pembrolizumab and atezolizumab (anti-PD-L1). In CheckMate-017 and 057, Grade 3 or 4 adverse events were less frequent with nivolumab than docetaxel—10 vs. 54% (057) and 7 vs. 55% (017). The most frequently reported adverse-events for nivolumab were fatigue (16% both), decreased appetite (10% in 057, 11% in 017) and asthenia (10% both). Frequency of select adverse events (thought to be immune-related in the nivolumab arm) for 057 (listed first) and 017 (listed second) compared with docetaxel (T) were rash (9% nivo v 3% T, 4% nivo v 6% T), hypothyroidism (7% nivo v 0% T, 4% nivo v 0% T), diarrhoea (8% nivo v 23% T, 8% nivo v 20% T) and pneumonitis (3% nivo v <1% T, 5% nivo v 0% T) [21,22]. In KEYNOTE-010 (pembrolizumab) and OAK (atezolizumab), grade 3–5 treatment-related adverse events occurred in 16% and 15% of the respective immunotherapy arms, compared with 35% and 43% in the docetaxel arms [24,26]. Notably, the nivolumab and atezolizumab second-line trials were unselected based on PD-L1 expression, whereas the pembrolizumab trial included patients whose tumours had PD-L1 ≥ 1%. This difference did not impact results, implying PD-L1 expression may not be important for response in the second-line setting.

Patients whose tumours harboured an *EGFR* mutation were the only subgroup not to benefit from immunotherapy compared with docetaxel in these trials. There was no overall survival benefit for *EGFR* mutation positive patients in CheckMate-057 (HR 1.18 (0.69–2.00)), KEYNOTE-010 (HR 0.88 (0.45–1.70)), or OAK (HR 1.24 (0.71–2.18)) [22,24,26]. Notably, IMpower-150 included a small number of TKI-naïve patients with EGFR-mutation, and did show a benefit of PD-L1-inhibition in the first-line [27]. This reduced responsiveness in second-line may relate to the lower TMB observed in lung cancers with known driver mutations [28].

Phase III trials of vaccines in the second line setting have not been widely practice changing to date. The STOP trial tested a vaccine, belagenpumatucel-L (“Lucanix”), derived from 4 NSCLC cell lines transfected with a transforming growth factor-B2 (TGF-B2) antisense plasmid (to decrease expression of the immunosuppressive protein). The trial showed no difference in OS (20.3 v 17.8 m, HR 0.94, *p* = 0.594) and no difference in PFS (4.3 v 4 m, HR 0.99, *p* = 0.947) [29]. Racotumomab is an anti-idiotype antibody, meaning it recognizes and binds the variable region of another antibody. If the recognized antibody is specific to cancer, the anti-idiotype antibody can mimic the original cancer antigen and induce an anti-cancer immune response. Racotumomab is given as an intra-dermal injection every two weeks for two months, and then monthly on an ongoing basis. It has been tested in a single phase II/III trial as maintenance therapy following platinum-based chemotherapy, and was found to be tolerable and effective compared with placebo (median OS 8.23 v 6.8 months, median PFS 5.33 v 3.9 months) [30]. The drug is licensed for use in Cuba and Peru and further research is ongoing [31].

### 3.2. Metastatic Disease—First Line

Trials in the first-line setting showed unexpected discordance between pembrolizumab and nivolumab. The KEYNOTE-024 phase III assessed pembrolizumab compared with investigator choice platinum-based chemotherapy in patients with NSCLC, without targetable mutation and with PD-L1 immunohistochemical tumour expression of greater than or equal to 50% using the Dako platform (22C3 antibody) (30.2% of screened cases) [32]. The trial was positive, with the primary endpoint of median PFS of 10.3 months vs. 6.0 months and strongly significant hazard ratio (HR) for risk of progression or death of 0.5 (95% CI, 0.37–0.68; *p* < 0.001), and mOS of 30 vs. 14.2 months [32,33]. Additionally, there was less treatment toxicity in the pembrolizumab arm compared with chemotherapy. This led to accelerated FDA approval of the drug in the first-line setting for patients with tumours expressing PD-L1 ≥50% in October 2016 (www.fda.gov).

The analogous trial using nivolumab, CheckMate-026, was a negative trial [25]. In this trial, patients with NSCLC with PD-L1 immunohistochemical expression ≥1% (28-8 antibody) were recruited and randomised to receive nivolumab or investigator-choice platinum-based chemotherapy. The primary end-point of PFS among patients with PD-L1 expression ≥5% was not met; median PFS was 4.2 months within the nivolumab arm vs. 5.9 months within the chemotherapy arm, with a HR for progression or death of 1.15 (95% CI, 0.91–1.45; *p* = 0.25) [25]. The median OS was similar for both groups (14.4 vs. 13.2 months) and the immunotherapy was better tolerated. There was also no improvement in PFS in a post-hoc sub-group analysis of those with PD-L1 expression ≥50%.

Much post-hoc analysis has been made into why the trial was negative. Imbalances in the baseline disease and patient characteristics were thought to have contributed significantly-there were more females (45 vs. 32%) and more favourable disease characteristics (median sum target lesions 68 vs. 82 mm, liver metastases 13 vs. 20%) within the chemotherapy arm. Exploratory analysis showed the chemotherapy arm had more patients with high TMB than the nivolumab arm, and a subgroup analysis of patients with high TMB showed median PFS significantly improved in the nivolumab arm (9.7 months vs. 5.8 months). There remains no evidence for checkpoint inhibitor monotherapy in the first-line setting for patients whose tumours have PD-L1 expression ≤50%. There is however strong evidence for combination CPIs, either with each other or with chemotherapy, in this group (discussed below, Combination Therapy). 

### 3.3. Adjuvant Treatment

Localised lung cancer has a poor prognosis-the five-year survival following complete resection of stage IIIA disease is 36%, and this figure approaches 20% in unresectable stage IIIB disease treated with chemoradiotherapy [34]. These figures have not changed since the introduction of adjuvant chemotherapy in 2004 [35]. Three major phase III trials that have applied immunotherapy in this setting are MAGRIT, START and PACIFIC.

MAGRIT screened over 13,000 patients with resected NSCLC and randomised over 2000 to receive placebo or vaccination with MAGE-A3 immunotherapeutic, comprising recombinant MAGE-A3, a tumour antigen commonly overexpressed in lung cancer, fused to an immunostimulant [36]. Adjuvant treatment with MAGE-A3 did not show a treatment effect compared with placebo, leading to cessation of further development of the compound in NSCLC.

Tecomotide (L-BLP25) is a vaccine against MUC1, a glycoprotein that is aberrantly glycosylated and overexpressed in NSCLC, and thought to play a role in proliferation and survival of cancer cells. Early phase trials of the drug showed improvement in OS when given following chemo-radiation for unresectable stage III NSCLC, however the multinational phase III trial, START, showed no difference in OS compared with placebo [37]. Further investigation of the subgroup that appeared to benefit, those receiving concurrent (rather than sequential) chemoradiation, did not support the subgroup analysis and clinical development of the drug was discontinued in 2014.

In contrast, PACIFIC has proved a practice changing trial based on strongly positive interim results. In this trial, one year of durvalumab, a blocking antibody to PD-L1, was compared to placebo as consolidation therapy following chemoradiation for unresectable stage III NSCLC [38]. The co-primary endpoints were PFS and OS, the first of which was reported in the interim analysis in November 2017. Median PFS in the durvalumab arm was 16.8 months, compared with 5.6 months in the placebo arm, with a HR for disease progression or death of 0.52 (95% CI, 0.42–0.65, *p* < 0.001) and 18 month PFS rate of 44.2% vs. 27.0% [38]. This benefit came with significant risk of pneumonitis—3.4% of patients on the durvalumab arm developed grade 3 (severe symptoms) or 4 (life-threatening) events vs 2.6% in placebo. Based on this data, the FDA has approved the use of durvalumab in unresectable stage III NSCLC patients who have not progressed following definitive chemoradiation.

### 3.4. Combination Therapy

Combination therapy has the potential to address overlap within immune tolerance pathways and avert resistance to checkpoint inhibitor monotherapy; it appears likely that combinations will improve response rate and durability. Dual checkpoint blockade with CTLA-4 and PD-1/PD-L1 blocking agents has established efficacy, presumably due to synergism of blocking T-cell inhibition at two phases of activation. A similar logic can be applied for blockade of novel inhibitory checkpoints, including indoleamine 2,3 dioxygenase (IDO) and lymphocyte activation gene 3 (LAG-3). Immunotherapy combined with chemotherapy may provide synergy through mechanisms such as reducing immunosuppressive cell activity, inducing PD-L1 expression and increasing tumour antigen cross-presentation [39]. VEGF is another potentially synergist target. Excessive VEGF production in cancer leads to abnormal TME vasculature and lymphocyte trafficking, facilitating tumour immune-evasion—blocking this pathway may thus improve checkpoint inhibitor efficacy. Conversely, VEGF-inhibitors promote vessel normalisation in a Th1-dependent reaction, which may be improved by skewing towards this subtype through PD-1/PD-L1 blockade [40,41]. Furthermore, upregulation of PD-L1 has been found in bevacizumab-refractory tumours and may be an escape mechanism [40].

CheckMate-012 assessed nivolumab combined with ipilimumab in a phase I setting in first line NSCLC [42]. It showed a response rate of 47% for all patients and 57% amongst those with tumour PD-L1 expression >1%. This combination was tested in the phase III setting in CheckMate-227, with the co-primary endpoint modified to include TMB “high” patients only (>10 non-synonymous mutations/megabase). Of patients evaluable for TMB, 45% were TMB high and, in this group, the combination was superior to platinum-based chemotherapy with a one year PFS rate of 42.6% vs. 13.2%, median PFS of 7.2 vs. 5.5 months and a HR for progression or death of 0.58 (95% CI, 0.41–0.81) [43]. Benefit was seen independent of PD-L1 expression, and toxicity was comparable to the control arm, with rate of grade 3 or 4 events of 31.2% vs. 36.1% with chemotherapy [43].

The MYSTIC trial is the equivalent for the Astra-Zeneca compounds, durvalumab and tremelimumab (anti-CTLA-4), in patients whose tumours express PD-L1 ≥ 25% by the VENTANA platform (SP263 antibody). Co-primary endpoints are PFS and OS, and it was announced in July 2017 that the drug combination had not met the PFS endpoint. The final analysis of OS is expected in the second half of 2018. Based on the experience from the melanoma cohort and the CheckMate-data in lung, it does appear that combination therapy can provide a broader, more durable response [44]. Should the final MYSTIC data not meet the OS end-point, doubt will likely be cast on the efficacy of tremelimumab, an IgG2 antibody with slightly different binding properties to its IgG1 counterpart, ipilimumab, rather than the potential of combination therapy [45].

KEYNOTE-189 is the phase III follow-on from the positive phase II KEYNOTE-021 Cohort-G, comparing pembrolizumab or placebo combined with platinum doublet chemotherapy in the first-line setting in non-squamous/non-EGFR/ALK pathology. First line pembrolizumab with chemotherapy was superior to standard chemotherapy for all reported endpoints and across all subgroups, including in tumours with PD-L1 expression <1% [46]. The estimated survival rate at 12 months (with 10.5 m median follow-up) was 69.2% in the pembrolizumab-combination arm and 49.4% in the chemotherapy arm, and the median PFS was 8.8 vs. 4.9 months. There were similar grade 3 or higher adverse events in both groups (67.2% and 65.8% respectively), with a notable exception of acute kidney injury (mostly nephritis), which occurred in 5.2% in the pembrolizumab arm (G3-5: 2%) compared with 0.5% in the chemotherapy-alone arm (G3-5: 0%) [46,47]. 

IMpower-150 is a phase III trial combining atezolizumab and chemotherapy (carboplatin/paclitaxel) with bevacizumab, a VEGF inhibitor. It was announced at ESMO IO in 2017, with further data at AACR 2018, that the co-primary endpoint of PFS had been met, with a median PFS of 8.3 months compared with 6.8 months and a HR of 0.61 (95% CI, 0.52–0.74) [27,48]. Notably, this trial included patients with EGFR and ALK-driven tumours—a similar benefit was seen in these groups, as well across all PD-L1 expressions [27].

Two major hurdles that combination regimens must overcome are efficacy and tolerability—the ideal treatment being one that would trigger specific anti-tumour immunity without auto-immunity. There are currently over 170 active interventional studies using immunotherapy in NSCLC listed on clinicaltrials.gov, many combining immune therapies with other immune therapies, chemotherapy or radiotherapy. Novel targets in the phase III setting include the enzyme IDO, an ancillary checkpoint that promotes an immunosuppressive TME by depleting the essential amino acid tryptophan. Response rates to anti-PD-1 plus anti-IDO1 appeared higher than with anti-PD-1 alone in early trials, however, the pivotal phase III trial in melanoma failed to meet its endpoint and several large trials, including those for NSCLC, were subsequently scaled back or halted [49].

### 3.5. Current Clinical Practice

Chemotherapy is an effective treatment for NSCLC and remains a vital part of treatment for the majority of patients. Evidence for immunotherapy in NSCLC however is increasing, and its indications are broadening. The National Comprehensive Cancer Network (NCCN) guidelines released in February 2018 include immunotherapy recommendations in adjuvant, first- and second-line settings [50]. In metastatic disease, NCCN-endorsed first-line options include single agent pembrolizumab (PD-L1 ≥ 50%, no targetable mutations), or combination therapy with pembrolizumab/platinum-pemetrexed (non-squamous histology, all PD-L1 expression) based on KEYNOTE-189 [46,50]. Second-line recommendations include single-agent pembrolizumab (PD-L1 ≥ 1%), nivolumab or atezolizumab (all PD-L1) [50]. In the adjuvant setting, durvalumab is recommended following definitive concurrent chemo-radiation for inoperable stage II and III disease [50].

Globally, immunotherapy combination regimens in NSCLC are available through clinical trials, and participation in a clinical trial is encouraged for all eligible patients. The majority of combination trials use an anti-PD-1/PD-L1 backbone, partnered with either a second CPI (e.g., anti-CTLA-4) or chemotherapy, radiotherapy or alternative immunotherapy such as a vaccine or novel checkpoint-targeting drug (clinicaltrials.gov). With expanding therapeutic targets comes expanding toxicity, which can be unpredictable and severe. Treating clinicians must be adept at recognizing and treating auto-immune toxicity early, which can often prevent progression to more severe events.

## 4. Conclusions

Prior to immunotherapy, treatment for advanced NSCLC had not changed significantly since the broad uptake of chemotherapy over best supportive care in the mid 1990s [51]. In the adjuvant setting, surgical and radiotherapy techniques had improved, but there had been no significant change in systemic treatment since cisplatin-based chemotherapy was shown to improve outcomes in the early 2000s [35]. The introduction of immunotherapy in NSCLC has marked a turning point in treatment of the disease. Practice-changing clinical trials have been abundant in recent years, and checkpoint inhibitors have become the standard of care in advanced disease, and will likely soon be standard in some adjuvant settings.

Systemic treatment for NSCLC is now rapidly evolving with significant advances for patient outcomes. Immunotherapy is effective and well-tolerated in a proportion of patients; the goal now is to broaden the efficacy and durability of the drugs. Combination therapies are likely to achieve this goal, potentially at the cost of higher toxicity. Many large international trials testing combination treatments are ongoing—it is likely that standard treatment will continue to evolve significantly over the coming years.

## Figures and Tables

**Table 1:** Immunotherapy in Non-small cell lung cancer—Phase III trial results. Negative results in bold.

2nd/3rd Line Metastatic NSCLC				
	Population	Arms	Results	Reference
KEYNOTE-010 NCT01905657	All histologies PDL1 ≥ 1%	Pembrolizumab 2 mg/kg Pembrolizumab 10 mg/kg Docetaxel	mOS (PDL1 ≥ 1%): 10.4 (2 mg/kg) v 12.7 (10 mg/kg) v 8.5 m mOS (PDL1 ≥ 50%): 14.9 v 17.3 v 8.2m **mPFS 3.9 v 4.0 v 4.0 m**	Herbst et al. Lancet 2016; 387: 1540–1550
CheckMate-017 NCT01642004	Squamous All PD-L1	Nivolumab Docetaxel	mOS: 9.2 v 6.0 m 1Y OS: 42 vs 24%	Brahmer et al. New Engl J Med 2015; 373: 123–135
CheckMate-057 NCT01673867	Non-squamous All PD-L1	Nivolumab Docetaxel	mOS: 12.2 v 9.4 m 1Y OS: 51 v 39%	Borghaei et al. New Engl J Med 2015; 373: 1623–1639
STOP NCT00676507	All histologies No PD on 1L platinum	Maintenance belagenpumatucel-L Placebo	mOS 20.3 v 17.8 m, HR 0.94, *p* = 0.54 PFS 4.3 v 4.0 m	Giaccone et al., Eur J Cancer, 2015; 51(16): 2321–2329
OAK NCT02008227	All histologies All PD-L1	Atezolizumab Docetaxel	1Y OS: 55 v 41% 18m OS: 40 v 27%	Rittmeyer et al. Lancet 2017; 389:255–265
**1st line metastatic NSCLC**				
	**Population**	**Arms**	**Results**	**Reference**
KEYNOTE-024 NCT02142738	All histologies PD-L1 ≥ 50%	Pembrolizumab 200 mg Platinum-Doublet → pembro on PD	mOS: 30 v 14.2 m mPFS: 10.3 v 6.0 m	Reck et al. New Engl J Med 2016; 375: 1823–1833 Brahmer et al. WCLC 2017 abstract OA17.06
CheckMate-026 NCT02041533	All histologies PD-L1≥ 1%Endpoints analysed on PD-L1 ≥ 5%	Nivolumab 3 mg/kg Platinum-Doublet	**mPFS: 4.2 v 5.9 m** **mOS: 14.4 v 13.2 m** **(HR 1.02, CI 0.8–1.3)**	Carbone et al. New Engl J Med 2017; 376: 2415–2426
KEYNOTE-189 NCT02578680	Non-squamous or NOS All PD-L1	Pembrolizumab 200 mg q3w (2 years) + 4 cycles pemetrexed + carboplatin AUC5 (PC) 4 cycles PC → pembro on PD	OS@1Y 69.2% v 49.4% mPFS 8.8 v 4.9 m	Gandhi et al., New Engl J Med 2018: Presented at AACR 2018.
CheckMate 227 NCT02477826	Non-squamous All PD-L1 All TMB—**endpoint assessed in HI TMB only	Nivolumab/Ipilimumab Nivolumab Platinum doublet chemotherapy	mOS 23 v 16.4 m mPFS 7.2 v 5.5 m OS@1Y: 67 v 58% PFS@1Y: 42.6% v 13.2%	Hellman et al, New Engl J Med 2018: Presented at AACR 2018
IMpower-150 NCT02366143	Non-squamous All PD-L1 Included EGFR/ALK patients	Atezolizumab + CP → atezo maintenance Atezolizumab + CP + bevacizumab → atezo + bev maintenance CP + bevacizumab → bev maintenance	B v C: mPFS: 6.8 v 8.3 m prelim mOS (immature): 19.2 v 14.4 m	Reck et al., ESMO IO 2017 Kowanetz et al., AACR 2018
**Adjuvant NSCLC**				
	**Population**	**Arms**	**Results**	**Reference**
PACIFIC NCT02125461	Stage III unresectable NSCLC Post chemoradiation All PD-L1	Durvalumab 10 mg/kg q2w for up to 12 m Placebo	12 m PFS: 55.9 v 35.3% 18 m PFS: 44.2 v 27.0%	Antonia et al, New Engl J Med 2017; 377: 1919–1929
START NCT00409188	Unresectable stage III NSCLC Post chemoradiation	Tecomotide (T) q1w for 8 w, then q6w until PD Placebo (P), as above	**mOS 25.6 v 22.3 m**	Butts et al., Lancet Oncology 2014; 15(1): 59–68
MAGRIT NCT00480025	Completely resected stage I-IIIA NSCLC	IM recMAGE-A3 with AS15 immunostimulant placebo	**Median DFS 60.5 v 57.9 m**	Vansteenkiste et al, Lancet Oncology 2016; 17(6): 822–835

**Table 2:** Immunotherapy in NSCLC—Upcoming phase III trials.

Metastatic Trials		
Nivolumab	CheckMate-9LA NCT03215706	1L NSCLC Nivo + ipi + chemotherapy v chemotherapy
Pembrolizumab	KEYNOTE-598 NCT03302234	1L NSCLC, PD-L1 ≥ 50% Pembro + ipi v pembro
	KEYNOTE-042 NCT02220894	1L NSCLC, PD-L1 ≥ 1% Pembrolizumab v SoC in Strong (PD-L1 ≥ 50%) v weak (PD-L1 1–49%) staining tumours
	KEYNOTE-715 NCT03322566	1L NSCLC, all PD-L1 IDOi + pembro + chemo v IDOi + pembro v placebo + pembro + chemo
	KEYNOTE-407 NCT02775435	1L squamous NSCLC, all PD-L1 Pembro + carbo-paclitaxel/nab paclitaxel v carbo-paclitaxel/nab paclitaxel
Durvalumab	POSEIDON NCT03164616	1L NSCLC Durva + tremelimumab (treme) + chemotherapy v chemotherapy
	MYSTIC NCT02453282	1L NSCLC Durva + treme v durva v chemotherapy
	NEPTUNE NCT02542293	1L NSCLC Durva + treme v chemotherapy
	ARCTIC NCT02352948	3L NSCLC A: PD-L1+ tumours durva vs SoC B: PD-L1—tumours durva v durva + treme v treme v SoC
Atezolizumab	IMpower-130 NCT02367781	1L non-squamous NSCLC Atezolizumab + Abraxane v Abraxane
	IMpower-131 NCT02367794	1L squamous NSCLC Atezolizumab + carbo/taxol v atezo + carbo/Abraxane
	IMpower-132 NCT02657434	1L non-squamous NSCLC Atezolizumab + platinum/pemetrexed v platinum/pemetrexed
	IMpower-110 NCT02409342	1L NSCLC, PD-L1 ≥ 1% Atezolizumab v platinum-doublet
	IMpower-111 NCT02409355	1L squamous NSCLC, PD-L1 ≥ 1% Atezolizumab v platinum-doublet
Avelumab	Javelin-100 NCT02576574	1L NSCLC, PD-L1+ Avelumab v platinum-doublet
	Javelin-200 NCT02395172	2L NSCLC, PD-L1+ Avelumab v docetaxel
Racotumomab	NCT01460472	Maintenance following 1L treatment Open label v best supportive care
**Adjuvant trials**		
Nivolumab	ANVIL NCT02595944	Nivo (1y) v no treatment
Pembrolizumab	KEYNOTE-091 NCT02504372	Pembro (1y) v placebo
Durvalumab	NCIC BR31 NCT02273375	Durva (1y) v placebo
Atezolizumab	IMpower-010 NCT02486718	Atezo (48 weeks) v no treatment All patients receive chemo
